# Early posterior negativity indicates time dilation by arousal

**DOI:** 10.1007/s00221-020-05991-9

**Published:** 2020-12-05

**Authors:** Ezgi Özoğlu, Roland Thomaschke

**Affiliations:** Cognition, Action, and Sustainability Unit, Department of Psychology, Albert-Ludwigs University of Freiburg, Freiburg, Germany

**Keywords:** Time perception, Dilation of time, Temporal bisection, EPN, Arousal

## Abstract

We investigated whether Early Posterior Negativity (EPN) indicated the subjective dilation of time when judging the duration of arousing stimuli. Participants performed a visual temporal bisection task along with high-level and low-level arousing auditory stimuli, while we simultaneously recorded EEG. In accordance with previous studies, arousing stimuli were temporally overestimated and led to higher EPN amplitude. Yet, we observed that time dilation and EPN amplitude were significantly correlated and this effect cannot be explained by confounds from stimulus valence. We interpret our findings in terms of the pacemaker–accumulator model of human timing, and suggest that EPN indicates an arousal-based increasing of the speed of our mental clock.

## Introduction

Perceiving time is a crucial prerequisite for responding adaptively in our everyday lives. We need proper timing skills to follow traffic rules while driving, to cross a road as pedestrians, or to perform a musical piece. Although humans, like most species, possess the ability to time intervals with substantial accuracy, our time estimations are not perfect. Timing context or the properties of the timed event can lead to under- or overestimation. Well-established factors leading to such timing biases include, for example, environments requiring attention to secondary assignments (Macar et al. 1994), or time intervals marked by emotion-inducing stimuli (Droit-Volet and Meck 2007).

Systematic distortions of timing are commonly explained by the so-called Pacemaker–Accumulator model (PA, Buhusi and Meck 2005). According to the PA model, an internal clock processes temporal information through three stages: a clock, a memory, and a decision stage. A pacemaker generates pulses at a fixed rate. When we direct our attention to timing a currently running interval, the switch closes, and the accumulator stores these accumulated pulses. These pulses are then counted and–at the memory stage–compared with the typical number of pulses accumulated during a previously learned time interval. Based on this comparison, we make a temporal judgment about the interval just experienced: Is it relatively short or relatively long in relation to a previously learned interval?

From a pacemaker–accumulator (PA) model perspective, arousal and attention play a leading role in temporal distortions (Treisman 2013; Zakay and Block 1995) by manipulating the total number of pulses reaching the accumulator. According to the Attentional Gate Model, attentional resources can be divided between attending to external events and attending to time (Block and Zakay 1996). The switch component of the clock is responsible to process the external cues in the environment (regarding “temporal meaning of the stimulus) which refers to selective attention. And an attentional gate moderates the number of pulses reaching the accumulator in proportion to the attention internally allocated to timing. Arousal, on the contrary, is assumed to increase the rate of the pacemaker itself (while attention determines how narrow or wide the gate gets to allow those pulses through). Arousal is assumed to originate from different sources; such as autonomical arousal (e.g., physical changes as heart rate and galvanic skin response), wakeful arousal (being sleep or awake), and affective arousal (self-reports of experienced arousal; Satpute et al. 2019). Despite different sources, these measures are often found to be correlated with each other (Knyazev 2012; Jurysta et al. 2003), and neuro-imaging studies are suggesting an overlap of brain mechanisms involved in control of different types of arousal (Fuller et al. 2011; Parvizi and Damasio 2001). In empirical studies where the focus is to manipulate the experienced arousal, affective stimuli with varying properties (different emotional images or tones) are used to compare accompanying behavioral and physiological changes. When a to-be-timed interval is highly arousing—for example, emotional sounds (Noulhaine et al. 2007), facial expressions (Droit-Volet et al. 2004), emotional scenes (Angrilli et al., 1997), or electric shocks (Fayolle et al. 2015)—more pulses are generated in a unit of time and we perceive time as more extended than it actually is. This effect is typically referred to as "dilation of time."

A more recent conceptualization by Matthews and Meck (2016) has introduced the “processing principle” to explain the effect of non-temporal factors on subjective time. This principle suggests that the “strength” of stimulus’ representation (in the sense of being more vivid and clear), and the ease to extract information from this representation are leading to perceive the duration of that stimulus to be longer. In case of emotion—as a non-temporal feature of to-be-timed interval—emotionally more intense stimulus would create a stronger signal, whereas less intense, less salient stimulus would lead to a weaker signal with reduced temporal representation. Arousal as an emotional dimension is by definition the intensity of the emotional stimulus aspect. From this perspective, time stimulus presented with higher arousal should lead to a longer perception of duration. Similarly, Eagleman and Pariyadath (2009) proposed coding efficiency as an alternative to the internal clock models to explain subjective time variation. This account in general states that processes evoking larger neural activity would lead to a lengthened perception of time. For example, in oddball paradigms, the oddball stimulus is perceived to last longer than the common stimuli. According to the coding efficiency account, this effect is due to the mere repetition of a stimulus diminishing its neural response—also called repetition suppression. The coding efficiency assumes a direct correspondence between the magnitude of the neural response and subjective duration of the stimulus.

Yet, arousal effects on timing should not be regarded as distinct from attention, because (1) arousal is related to valence, and valence in turn affects attention, (2) arousal can enhance perception via attentional processing by prioritizing emotional stimulus over neutral ones (Vuilleumier 2015). In fact, the most prevalent models of emotion in the psychological literature conceive of arousal and valence as clearly distinct emotional factors. Specifically, the circumplex model of emotion (Russell 1980) assumes that each subjectively experienced emotion can unambiguously be described as a scalar product of its valence dimension and its arousal dimension. Yet, despite their descriptive independence, valence and arousal are strongly correlated in our everyday life. When a stimulus is highly arousing it is likely that it is also of strongly negative valence (Thomaschke et al. 2018). Or for example, with a negative emotion, such as a sense of threat, the stimulus is faster in capturing one’s attention and more difficult to disengage from (Cisler et al. 2009) as compared to positive ones. Similarly, positive stimulus is suggested to broaden the scope of attention (Frederickson and Branigan 2005), whereas negative emotions lead to narrowing down. On the other hand, high arousal stimuli can lead to greater behavioral and neural response, as emotional stimuli can lead to faster capture of attention and more-efficient processing via selective attention (Desimone and Duncan 1995; Vuilleumier 2015). For timing stimulus, this could mean stronger temporal representations of emotional time intervals compared to neutral ones.

Thus, in most everyday life scenarios, the lengthening effect in our subjective time is due to both increased arousal (clock speed and attention) and modulated attention by valence (attentional gate). Arousal can increase or decrease the amount of attention devoted depending on the type of evoked emotion which is a product of valence. And attention has a more direct effect on time dilation even in the absence of valence. More specifically, attention has four different aspects operating how we process information: working memory, competitive selection, top–down sensitivity control, and salience filter for significant stimuli (Knudsen 2007). Information kept in working memory is the base for our decisions and goal-directed behavior, and it can generate signals that improve the quality of information processing by enabling an overall lower level of signal-to-noise ratio in information processing. Yet, more important to our case, bottom–up processing of information depends on the salience of the stimulus—such as arousal level. Similar to what the processing principle proposes, salience filters are assumed to lead the nervous system to respond to salient stimulus with a stronger neural response—prioritizing the salient stimulus for working memory access as opposed to other competing stimuli. This way, the salient stimulus is perceived as “popping-out from the scene” (Egeth and Yantis 1997; Knudsen 2007). The effect of an emotional stimulus is therefore likely to be a combined one of arousal and attention.

For positive and negative emotions, the valence-based part of the effect is assumed not to be proportional to the experienced arousal. This is corroborated by findings that within the same level of arousal, for example, negative valence shows more pronounced time dilation effects than positive valence (Noulhaine et al. 2007). Similarly, emotions like threat or fear lead bisection functions to move leftward (as a psychometric sign of time dilation), whereas shame led to an opposite effect presumably due to redirection of attention (Droit-Volet and Meck 2007). The Evaluative Space Model (ESM; Cacioppo et al. 2011) explains affective experiences within distinct systems for processing positivity (appetitive motivation) and negativity (aversive motivation). The model also assumes an asymmetry in the activation patterns of positivity and negativity. Since aversive stimuli (avoiding danger) evoke stronger reactions for survival than appetitive stimuli (pursuing a mate); higher negativity results in more intense responses than higher positivity, which is referred to as “negativity bias” (Norris et al. 2010). Considering dilation of time, temporal distortions are assumed to be a result of increased clock speed combined with a narrowing of the attentional gate caused by the orientation of attentional sources. While most previous studies did confound manipulations of arousal with manipulations of valence, the present study aimed to focus primarily on arousal, and how its association with temporal distortions can be addressed independently of the valence or specifically negativity bias. Due to the above-mentioned close relationship between stimulus salience and working memory access, it is likely that valence and arousal have interaction effects on perception. Therefore, even manipulating only arousal can possibly affect attention allocation. However, as comparative approaches for different aspects of emotion are necessary to specify (at least to some extent) independent effects of arousal and valence, we aimed for a comparison that excludes an on-purpose valence manipulation. That way, the total effect of emotion would still be a byproduct of increased clock speed and attentional modulation. However, by avoiding the asymmetrical activation of positivity/negativity and its drastic effects on cognitive responses, the main source of the effect would be arousal.

Along with behavioral studies, neurophysiological findings, especially electrophysiological measures, have aided our understanding of human timing (for a review, see, e.g., Kononowicz et al. 2018). Recently, event-related potentials (ERP) were used to investigate also the emotional modulation of timing performance, as ERPs have proved useful to explain emotional processing in other cognitive domains, such as episodic memory (Johansson and Mecklinger 2003), working memory (Tays et al. 2011), or the processing of facial expressions (Grossman et al. 2007). One well-studied ERP component in emotional processing is the Early Posterior Negativity (EPN). EPN is an ERP that develops 200–300 ms after stimulus onset. It generates more negative values when selective attention is employed at emotional visual stimuli (Dolcos and Cabeza 2002; Schupp et al. 2004, 2006), and emotional auditory stimuli (Mittermeier et al. 2011; Jaspers-Fayer et al. 2012). As Schupp et al. (2006) suggest, this sensitivity stems from the intensity, which is the arousal component of emotion. Schupp et al. (2004) presented pleasant and unpleasant visual stimuli in two levels of arousal: high and low. Whereas unpleasant and pleasant images resulted in greater EPN values compared to the neutral category, high arousal stimuli also yielded greater EPN amplitude compared to low arousal stimuli within the same valence category. Therefore, though more frequently compared across different emotions that did not necessarily focused on arousal changes, EPN, indicates an arousal-based change with and without a valence component. Increased negativity for affective stimuli is assumed as selective processing of attentional allocation to motivationally more relevant stimuli. EPN has also been investigated in a timing context. In a recent study, Tamm et al. (2014) showed that EPN amplitude differed for positive and negative pictures in a time reproduction task. Specifically, erotic pictures yielded larger EPN amplitude than an aversive one, suggesting that selective early attention was more evident for positive stimuli. As they have compared high arousal negative images to high arousal positive and minimally arousal neutral images, from that study, it is not yet clear whether the EPN modulation by arousal is independent of the time dilation effect of arousal observed in other studies (see above), or whether both effects are somehow functionally related. If we consider EPN as a potential physiological signature of arousal-based time dilation, modulations in this component’s characteristics should be accompanied by changes in timing behavior as well, which is the relation explicitly targeted by the present study.

To our knowledge, previous studies on EPN, timing, and emotion have mainly focused on emotion in general, and used stimuli sets with varying levels of valence and arousal in a way where the dimensions were not independent (Tamm et al. 2014). Considering the high temporal resolution EEG provides, the current study focused on this idea and aimed at inducing an arousal-related behavioral change in timing behavior accompanied by systematic variation in EPN. Accordingly, in a temporal bisection paradigm, we expected time intervals marked with higher arousal stimuli to be perceived as longer, and to evoke larger EPN values than time intervals marked with lower arousing stimuli. Yet, contrary to previous studies, we controlled stimuli for valence. By controlling for valence, we increase the likelihood that any time dilation effect is due to arousal-based clock speed and attentional changes that is independent of valence-based strong physical and cognitive changes like negativity bias. If the expected EPN increase is correlated with time dilation, we can conclude that the EPN effect in time dilation is due to arousal-related clock speed and attentional changes.

## Experimental procedure

### Participant

Eighteen students with normal or corrected-to-normal vision at the Albert-Ludwigs University of Freiburg have participated. We discarded data from one participant due to excessive eye blinks and muscle artifacts. The final sample had seventeen participants (6 male, 15 right-handed, mean age (*M* ± *SD* years, 25.01 ± 2.7). Before participation, each participant signed an informed consent form and received 16 Euros.

### Procedure

Participants completed a temporal bisection task with simultaneous EEG recording. At the beginning of the procedure, we reminded participants to refrain from counting for the entire procedure. The task included a learning phase and a testing phase. The learning phase required participants to observe and memorize one short and one long anchor duration; 600–1900 ms, respectively. In each trial, these anchor durations were presented by the display duration of a blue square. Participants had to attend and learn how long the blue square remained on the screen. Each presentation was followed by informative feedback such as “This was the long/short anchor duration." Long and short anchors were each presented for ten times in a randomized order. Every participant completed the learning phase for one time.

**Fig. 1:**
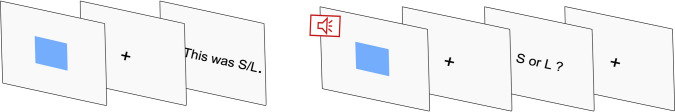
Sequence of a trial in the learning (on the left) in the testing part (on the right). See text for further details

After learning, participants completed two testing blocks with a three-minute break between them. The aim of the testing phase was to categorize various probe intervals in comparison to the learned anchor durations. The testing phase included seven different probe intervals. The spacing of these intervals was logarithmic between the short and the long anchor (i.e., 600, 727, 881, 1067, 1293, 1567, and 1900 ms). Each of these probe intervals was presented in a randomized order for 16 times in a block, resulting in 224 trials in total. In each trial, a probe interval was presented and asked for a comparison to the learnt anchor durations. The trial started with the presentation of probe duration. This presentation consisted of a blue square accompanied by a sound that had either high or low arousal levels. The sound presentation lasted through the entire probe interval. The probe interval was followed by a delay (randomly given between 1000 and 1500 ms) before participants could give their response. They were asked to decide whether the probe interval was closer to the short or long anchor. Participants indicated their response using the keys X or M on a standard QWERTZ keyboard for short and long, respectively. An inter-trial interval (ITI; randomly drawn between 1000 and 1500 ms) followed the response (see Fig. 1, for a schematic trial illustration). There was a 3-min break between the blocks. After the break, participants were reminded of the anchor durations by a one-time presentation of each anchor as in the learning phase.

Participants were seated at approximately 50 cm distance from a computer screen. Visual stimuli comprised a blue square (100 × 100 pixels). Auditory stimuli were taken from a validated battery of International Affective Digitized Sounds (IADS; Bradley and Lang 2007). These sounds were chosen based on their valence and arousal values (Please refer to Table 1 for affective and acoustic properties of these sounds). We choose tones in a way that they do differ as little as possible in obvious acoustical properties. Of course, it is, in principle, not possible to manipulate the arousal level of a tone without accompanying changes on many physical dimensions, especially “ecological” sounds, i.e., sounds typically occurring in our everyday surroundings. Considering the many characteristics of a sound—e.g., pitch, loudness, or timbre—“physically” equal tones would be fully identical and would consequently have an identical arousal level. The aim was to maintain an arousal manipulation while keeping the effect of valence constant for each condition. Therefore, the two sounds were expected to elicit high and low arousal while keeping a medium level of valence that is not necessarily positive or negative. Therefore, participants performed a visual timing task, where their performance is interfered by emotional sounds. Having two different levels of arousal, we intended to test comparatively how behavioral and physical changes results from low and high arousal conditions. Behavioral data collection was done by E-Prime (Psychology Software Tools, USA) software.

**Table 1:** Affective and acoustic properties of sounds

Sound (Nr)	Arousal	Valence	Lmax	Leq	Peak amp	Mean RMSD
High (704)	6.54	5.49	74.0	80.3	− 5.4	− 19.5
Low (698)	4.12	5.18	67.4	74.7	− 4.3	− 17.5

*L*_*max*_ maximum sound intensity, *Leq* equivalent continuous sound intensity, *Peak Amp* peak amplitude, *RMS* average root-mean-square power. Information is taken from Bradley and Lang (2007) and Yang et al. (2018)

### Data analysis

The proportion of long responses [p(long)] was calculated separately for low and high arousal stimuli. For each participant, a cumulative Gaussian function was fitted to these proportions across intervals (Coşkun et al. 2015). Accordingly, the point of subjective equality (PSE), the value for which the probability of long and short responses is equal [p(long) = 0.5], was estimated.

**Fig. 2:**
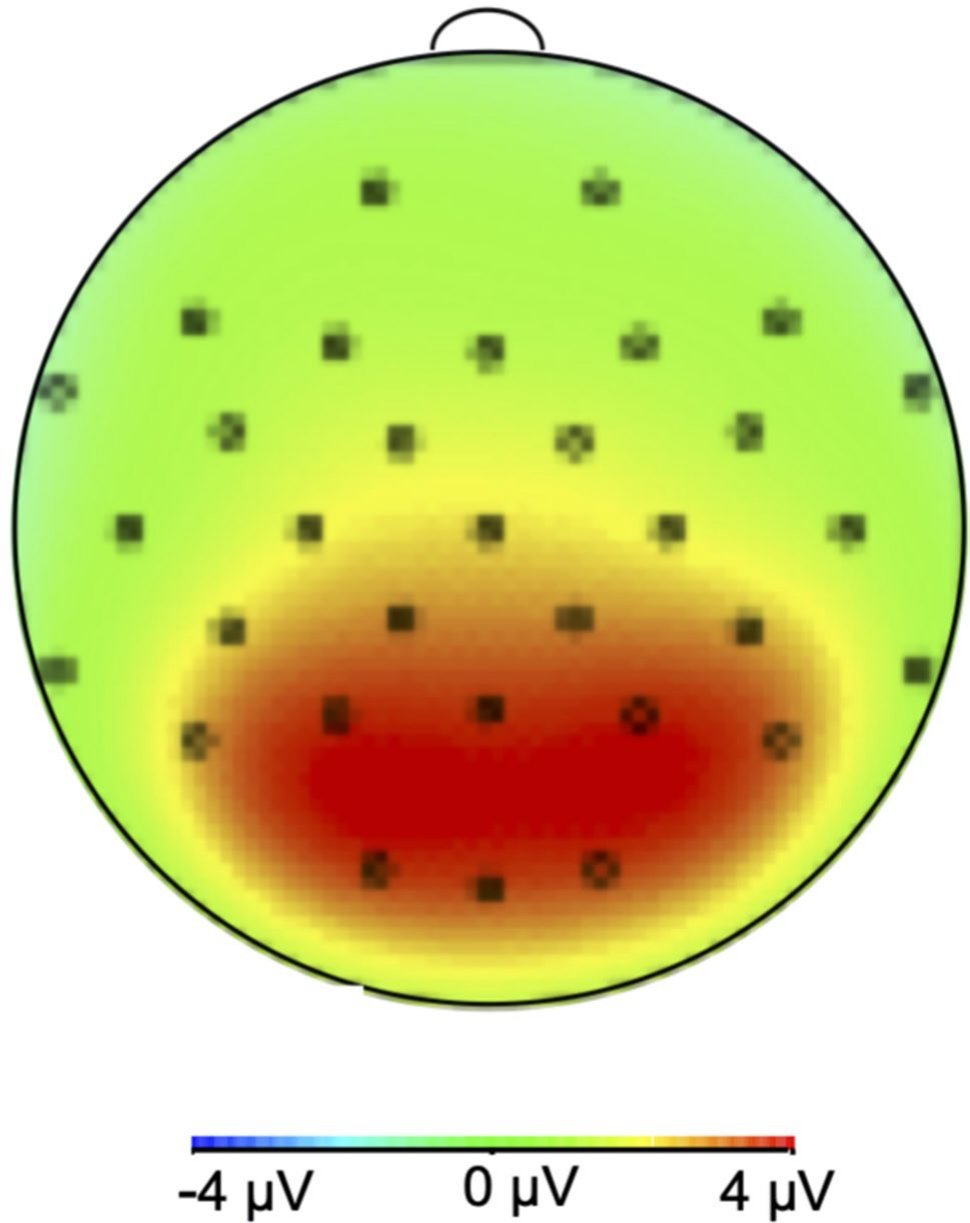
Voltage map during the analysis window of EPN (200–300 ms) averaged over participants, durations, and arousal levels

**Fig. 3:**
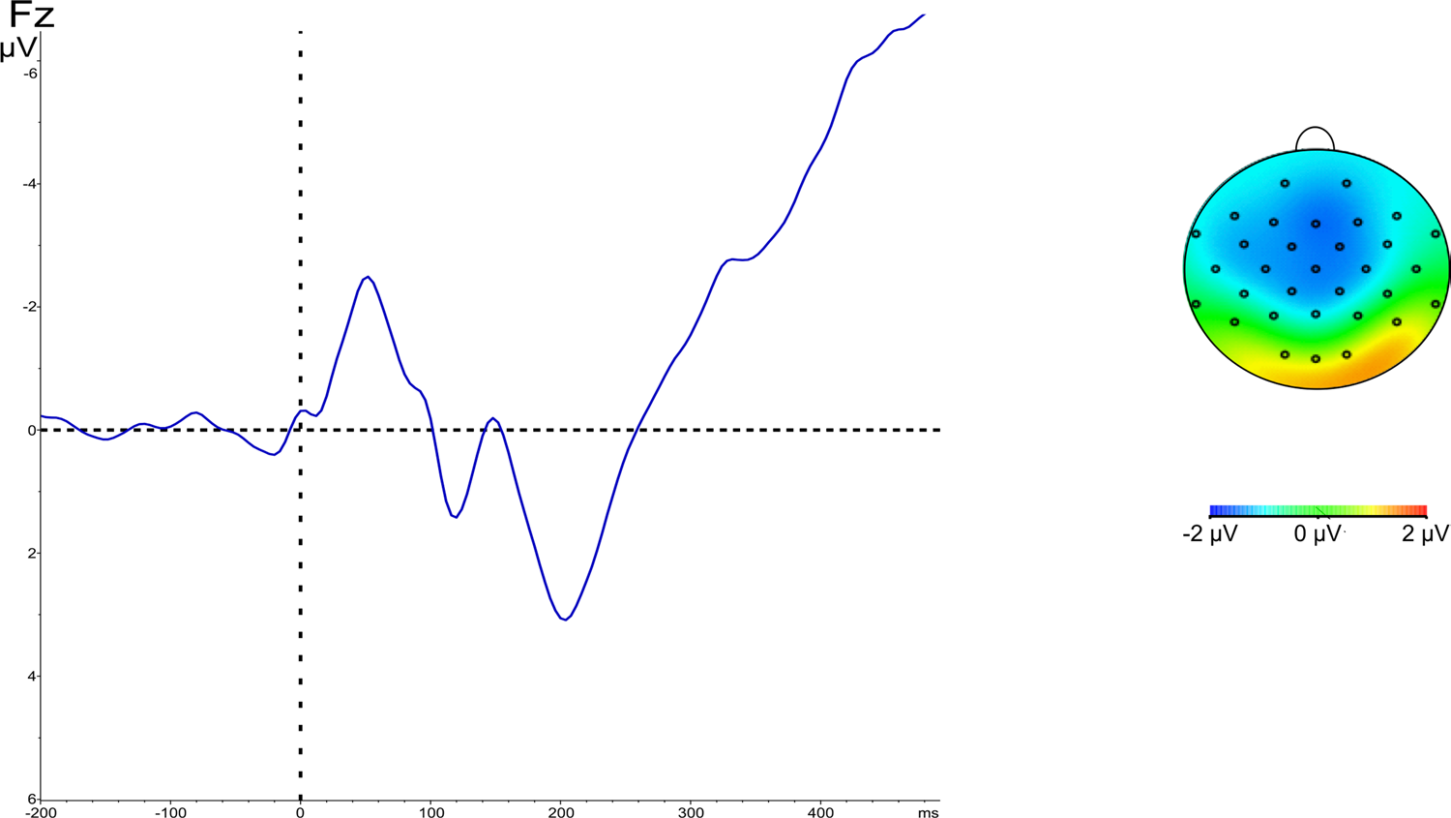
a. ERP of N1 from channel Fz, averaged over participants, durations, and arousal levels b. Voltage map of N1, between 16 and 80 ms

The EEG data were recorded using BrainVision Recorder software (Brainproducts, Germany) from 32 electrode locations (Fp1, Fp2, F3, F4, F7, F8, FT9, FT10, Fz, FC1, FC2, FC5, FC6, T7, T8, TP9, TP10, Cz, C3, C5, CP1, CP2, CP5, CP6, P3, P4, P7, P8, Pz, O1, O2, and Oz) with Ag/AgCl electrodes. A standard 10–20 layout was used. Electrode sites Fpz and Cz were used for ground and reference electrode, respectively. Vertical (above and below of the right eye) and horizontal (at the outer canthi of both eyes) electrooculogram (EOG) electrodes recorded the eye activity. The sampling rate was 500 Hz. Impedance was kept below 10 kΩ. BrainVision Analyzer software was used to analyze EEG data (Brainproducts, Germany). The EEG data were offline re-referenced to average of approximated mastoid electrodes Tp9 and Tp10; high-pass filtered to 0.01 Hz and low-pass filtered to 40 Hz. Ocular artifacts were corrected using independent component analysis (ICA). For ERP analysis; stimulus onset time-locked, 500 ms long epochs were created. A trial was excluded from further analysis when the change voltage exceeded 100 µV within sliding 200 ms windows. Overall, less than 5% of the data were discarded. The epochs were baseline corrected relative to 200 ms prior to the stimulus onset. EPN was calculated as the mean amplitude value between 200 and 300 ms after the stimulus onset (Schupp et al. 2004, 2006). ERP was calculated by averaging these epochs across trials. We included data from midline occipital (Oz), parietal (Pz), and central (Cz) sensors, along with bilateral parietal sensors from locations P3 and P4 based on the voltage map averaged over participants, duration, and stimulus type (Fig. 2). Based on grand average ERPs, we have observed N1 and included this potential in the analysis. N1 was observed between 60 and 120 ms in channel Fz (Fig. 3). For statistical analysis, we used the software R (R Core Team 2019) with lme4 (Bates et al. 2015). The minimum number of artifact-free trial was 24 for each arousal condition.

## Results

**Fig. 4:**
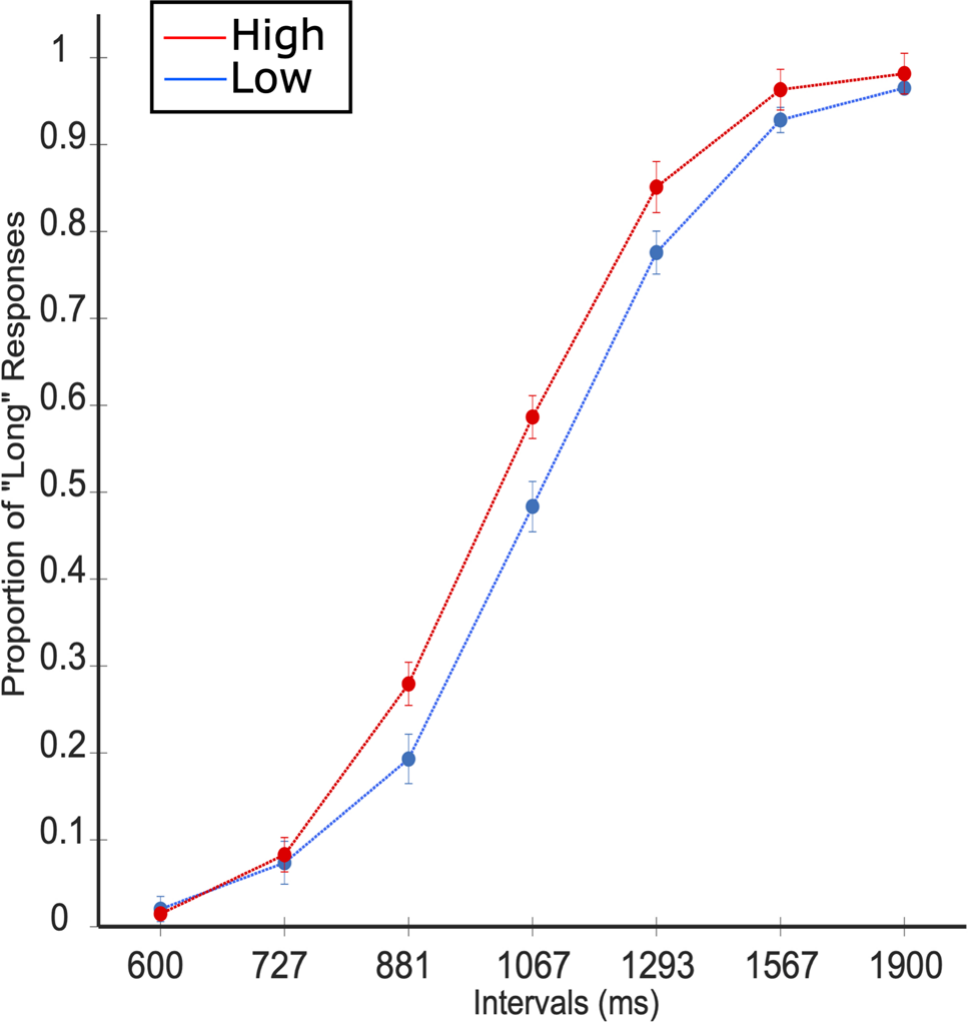
Temporal gradients for high and low arousal stimuli. Round markers refer to average proportions. Error bars stand for standard error

In behavioral timing performance as well as in EEG data, we looked at the contrast between low and high arousal conditions. As Fig. 4 shows, on average, high arousal stimuli led to higher proportion of long responses. To assess whether the arousal manipulation resulted in a meaningful difference in behavior, we compared the PSEs within subjects between both conditions (Table 2). High arousal stimuli lead to a significantly lower PSE than low arousal ones; *t*(16) = 4.41, *p* < 0.01, *d* = − 1.07.

**Table 2:** Mean and standard deviation of PSE

Arousal		
	Mean	SD
PSE		
High	1070	133
Low	1035	135

**Fig. 5:**
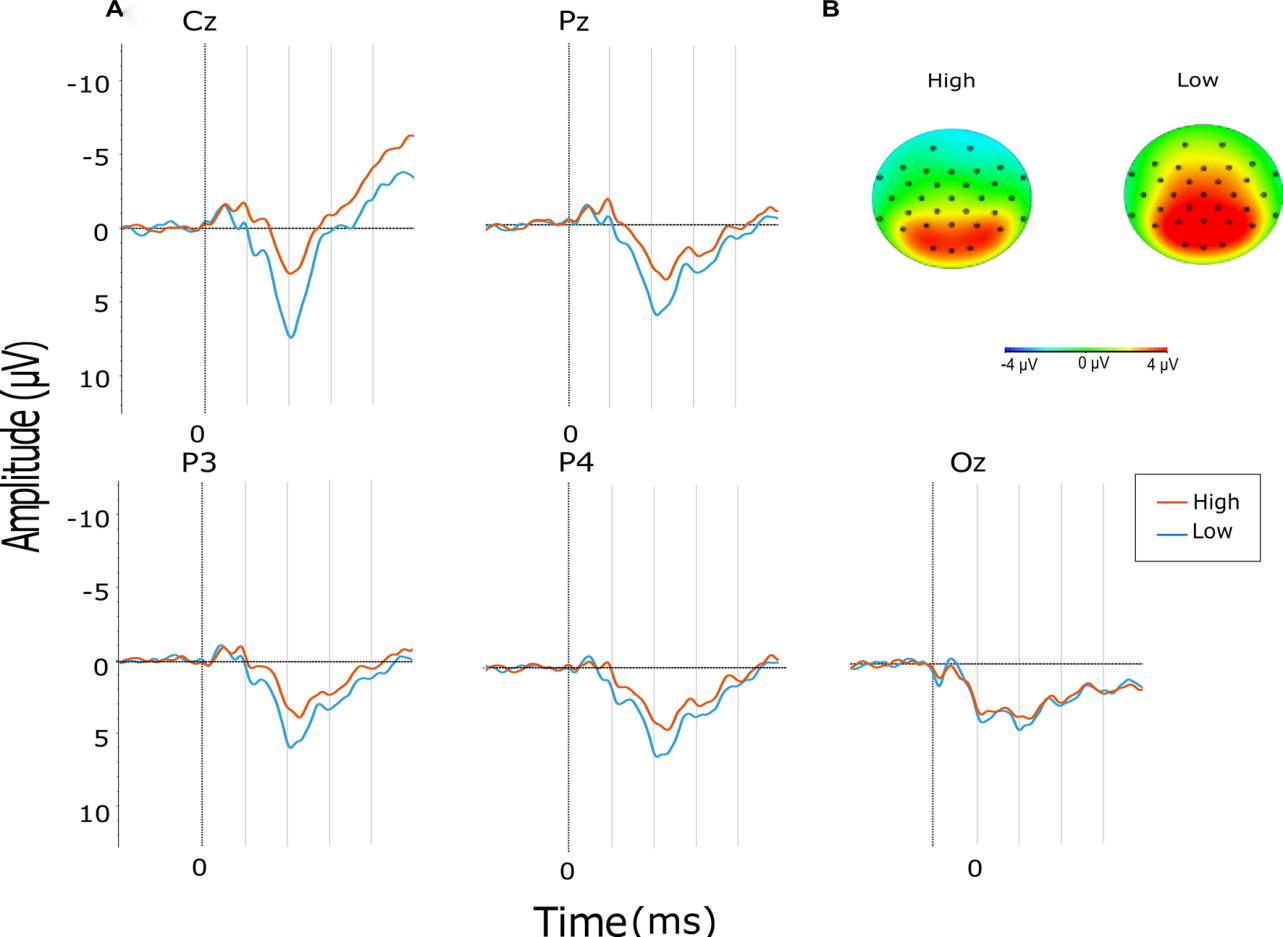
**a** Grand average of EPN for electrodes Cz, Oz, Pz, P3, and P4. Red and blue lines refer to high and low arousal stimuli, respectively. Time 0 refers to the interval onset. Vertical lines show 100 ms time steps. **b** Voltage maps of high and low arousal stimuli during the analysis window of EPN (200–300 ms)

We expected a similar difference between high and low arousal stimuli in ERP data (Fig. 5). Therefore, we constructed a linear-mixed effect model using the lmertest package in R (Kuznetsova et al. 2017), where EPN amplitude was the outcome variable, and duration, and arousal level (low vs high arousal sounds), electrode location (Cz, Pz, P3, P4, and Oz) were predictors. The model also included a random intercept per participant. The arousal level (*F*(1,1154) = 38.98, *p* < 0.001, *β* = − 1.17) was a significant predictor. There was no main effect of duration (*F*(1,1154) = 0.06, *p* > 0.05) and electrode location ((*F*(4,1154) = 1.66, *p* > 0.05). There was a significant interaction between electrode location and arousal level (*F*(4,1154) = 2.76, *p* < 0.05). There were no other significant interaction between arousal level and duration (*F*(1,1154) = 0.34, *p* > 0.05), duration and electrode location (*F*(1,1154) = 0.12, *p* > 0.05), or three-way interaction of predictors (*F*(1,1154) = 0.07, *p* > 0.05). Due to the significant interaction between arousal and electrode location, we conducted a simple effect analysis to establish the effect of stimulus type on different locations. Bonferroni correction was applied on multiple comparisons. High arousal stimulus led to greater EPN values on all electrode cites (for Cz, Fz, P3, P4, and Pz, β were − 1.87, − 2.00, − 0.96, − 0.85, − 1.15, respectively; all *p* < 0.001), except Oz (*p* > 0.05, Table 3).

**Table 3:** Mean and standard deviation of EPN

Channel	Arousal	Amplitude	
		Mean	SD
Cz			
High		1.04	3.76
Low		4.78	3.70
Pz			
High		1.83	4.09
Low		4.13	3.80
P3			
High		2.22	2.95
Low		4.15	3.00
P4			
High		2.84	3.01
Low		4.55	3.01
Oz			
High		3.18	2.91
Low		3.59	2.73

We have run a linear-mixed model on N1 amplitude with duration and arousal level as predictors, and with random effect given per participant. Neither of the predictors were found significant (for arousal, *F*(1208) = 0.97, *p* > 0.05: for duration, *F*(6208) = 0.44, *p* > 0.05, and there was no interaction effect (*F*(6208) = 1.78, *p* > 0.05). We have run the same model with N1 latency as outcome variable. While there was no effect of duration (*F*(6208) = 0.84, *p* > 0.05) or interaction (*F*(6208) = 0.98, *p* > 0.05), latency was predicted by arousal level (*F*(1208) = 5.82, *p* < 0.05, *β* = 3.18). Overall, high arousal stimuli led to earlier N1 potential than low arousal stimuli (Table 4).

**Table 4:** Mean and standard deviation of N1

Arousal	Amplitude		Latency	
	Mean	SD	Mean	SD
N1				
High	− 4.59	3.90	55.00	23.2
Low	− 4.31	4.03	49.10	23.5

**Fig. 6:**
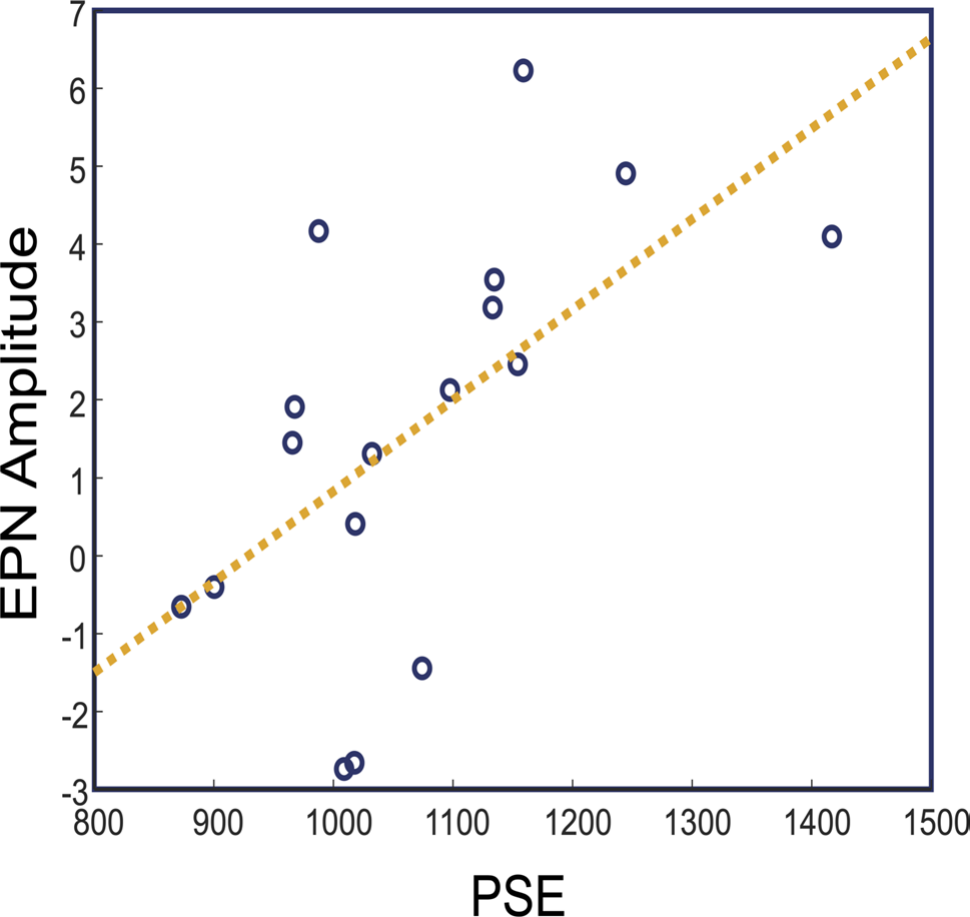
Correlation plot of PSE values (*x*-axis) and averaged EPN amplitudes for high arousal stimulus (*y*-axis)

If the shifts in PSEs stem from the same source that modulates EPN amplitudes, changes in behavioral data should be associated with changes in ERP data. Therefore, individual PSEs for high arousal stimuli are expected to positively correlate with the magnitude of average EPN amplitudes of high arousal stimuli (Fig. 6). A Pearson correlation between average EPN amplitude for high arousal stimulus and PSE for high arousal stimulus confirmed this expectation [*r*(15) = 0.66, *p* < 0.01], suggesting more negative amplitudes of EPN for reduced PSE values. We also ran a Pearson correlation test between PSEs from low arousal stimulus and averaged EPN values of low arousal stimulus, as well. Results showed a significant correlation, *r*(15) = 0.50, *p* < 0.05; suggesting there is a similar association between individual PSEs and magnitude of EPN amplitude for low arousal sounds. For both correlations, we have calculated Cook’s Distance for potential cases with high influence. Following Cohen et al.’s (2003) guideline, we have calculated the critical value of the F distribution at *α* = 0.50 with *df* = (k + 1, n-k-1). No cases were above the threshold in both correlations, suggesting no cases with high influence.

To further investigate the relationship between perceived time and EPN, we have conducted a trial-wise analysis on binary temporal responses (short/long) and EPN amplitude. We constructed a mixed-logistic regression with response as outcome variable, and EPN amplitude as predictor. Random effect was given for participant and for electrode location. EPN amplitude showed a significant effect on response *(β* = 0.99,* p* < 0.05*)*, as responding “long” led to lower (greater negativity) EPN amplitude.

## Discussion

Arousal-based distortions in our perception of time have previously been reported by behavioral studies. However, the neurophysiology of arousal-based time dilation is not yet well studied. In the current study, we aimed to establish an electrophysiological marker of arousal-based dilation of time. We employed a temporal bisection task using stimuli with two different arousal levels while keeping valence on a similar level. In this way, we aimed at investigating the time lengthening effect by arousal, which is typically associated with changes in clock speed, while avoiding a time lengthening effect by valence—which is typically associated with changes in an attentional gate aperture. We asked whether EPN (an ERP found in emotional information processing) is related to arousal-based changes.

Our data show that high arousal stimuli led to a leftward shift in the psychometric function compared to low arousal stimuli. PA models assume arousal to increase the speed of the internal clock; in line with this view, we regard the observed change to reflect an increased clock speed along with the processing of arousing stimuli through salience. As we controlled for stimulus valence, our findings are not likely to reflect a valence-induced narrowing of the attentional gate or asymmetrical changes in experienced emotions through negativity bias.

We also found a significant difference in EPN amplitudes for high and low arousal stimuli. An initial interpretation of such a finding was that EPN reflects early selective processing of stimulus based on its perceptual characteristics favoring arousing stimulus for further processing. Yet, our study shows that intervals presented with higher arousal sounds led to a differential processing of temporal information which was significantly correlated with a higher EPN amplitude. The association between the leftward shifted PSE values and greater negativity suggests that EPN is related to arousal-based time dilation. Our finding is in line with literature showing EPN amplitude to be sensitive to differences in stimulus complexity, picture content, valence, and stimulus size (Löw et al. 2005; De Cesarei and Codispoti 2006).

A key finding of our study is the correlation between PSE values and EPN amplitudes. In addition to the overall early occurrence of PSEs for high arousal stimulus, we tested by assessing individual differences whether there is an association between PSE points and EPN. If the EPN deflected to the extent that the participant was affected by the arousal, on an individual level, PSE should be lower for greater deflections and later for smaller deflections. This shows that both behavioral and electrophysiological modulations by arousal depend on how much the participant is affected by the stimulus. Single-trial analysis findings showed greater negative amplitudes for “long responses”, further supporting the link between EPN and timing behavior. We conclude that EPN is either a marker for arousal induced time dilation, or alternatively it might be governed by the same processes that control our timing mechanism under the effect of arousal. From a PA model perspective, our findings show that EPN is related to increasing clock speed.

From the processing principle perspective (Matthews and Meck 2016), such a relationship between EPN deflections was expected for reduced PSEs, since EPN is sensitive to affective stimuli—which also makes it more salient. Assuming that salience of stimulus (maintained by the arousal level) is enabling more vivid temporal representations: the greater the EPN deflection, the more dilated perception should be observed. Considering the coding efficiency approach (Eagleman and Pariyadath 2009), as much as the arousal affects the person, the neural response should be greater to the same extent (similarly, the same stimulus might evoke a smaller neural response if it was not received as highly arousing). The association between EPN deflection and PSE can be reflecting the mapping between neural response to arousal and dilated time effect in response to the arousal. Therefore, adaptation of sensory responses can be underlying the lengthened time, which has been previously suggested for duration distortions of sub-second visual stimuli (Sadeghi et al. 2011).

Even though studies on neural underpinnings of EPN are not fully converging, findings generally disagree with the involvement of dopamine oscillation. When dopamine levels were manipulated, increased or decreased levels of dopamine did not affect the EPN amplitude to affective stimuli (Franken et al. 2008). There seems to be no strong evidence to link EPN changes to the dopamine-based theory of timing. However, norepinephrine has been theoretically linked to subjective time as it is closely associated with pupil dilation (Joshi et al. 2016; Murphy et al. 2014) and pupil dilation has been correlated with subjective time (Suzuki et al. 2016; Faber 2017). Another link of norepinephrine was through a certain posterior positive wave, namely P3. In a temporal oddball task, Ernst et al. (2017) have found that the overestimation of target oddballs was associated with greater amplitude of posterior P3. This component has been previously shown to reflect the neuromodulatory locus coeruleus–norepinephrine system, a correlate of decision-making that involves motivationally relevant stimuli (e.g., arousal; Aston-Jones et al. 1994; Nieuwenhuis and Aston-Jones 2005). When compared to each other, EPN and P3 seem to belong to distinct processes as they have different time courses (posterior P3 occurs relatively later and develops slower), though they are both linked to arousal-related stimulus processing. Considering its role in targeting selective attention to emotional stimuli, EPN can be tagging emotionally relevant (or more generally addressing the “salient”) stimulus to facilitate its processing to create more vivid representations in further stages. Such temporal representations could eventually lead to the subjective experience of time, where the magnitude of the overestimations depends on the arousal effect—or EPN deflections. In this case, EPN can be a marker of subjective time, but the overall effect of emotion can involve separate phases where dopamine- and norepinephrine-based changes are more pronouncedly reflected in later stages of stimulus processing. Thus, we suggest that the dopaminergic mechanisms and the EPN-related mechanisms investigated in the present study are related to different components of time dilation.

EPN studies have been commonly conducted with visual stimulation (Blechert et al. 2012; Weinberg and Hajcak 2010) where EPN has been observed as a negative deflection over the occipito-parietal region, and its amplitude shows more pronounced deflection to emotional compared to neutral stimuli. In the current study, we observed more a central pattern of EPN possibly due to the use of auditory stimulation (Grass Bayer and Schacht 2016). Nevertheless, EPN exhibited emotion-sensitive characteristics. Yet, should be noted that there characteristics are not identical to the EPN typically found within purely visual stimuli. One possible interpretation is that visual and auditory stimulations may have separate effects on ERPs leading to an inhibitory response in the modality to perform the task in. We found an earlier N1 component for high arousal sounds, which could be due to the effect of arousal on visually evoked potentials (Vogel and Luck 2000). For future studies, using a control modality can be useful to account for any modality-based changes in ERPs.

An important limitation of the interpretability of our study is related to the use of ecological tones. In this study, we used two tones that are similar in valence and differing in arousal levels, taken form the battery of IADS. This battery provides a standardized approach to manipulate arousal and valence while using ecological sounds—e.g., sounds we are exposed in everyday environments. Ecological sounds enable a semantic meaning to the presented stimulus, which creates an emotional effect that cannot be mainly explained by its physical properties (Tajadura-Jiménez and Västfjäll 2008). Yet, this comes with the disadvantage that we cannot completely rule out the difference between acoustical characteristics of the sounds as sources of arousal well. Unfortunately, a systematic parametric control of tones (like sine tones with different pitch) would deprive tones of any ecologic meaning and thus of their potential to elicit high or low arousal in a controlled way. Thus, we recommend for future studies to employ different ecological sounds with different acoustic properties, to increase generalization. In summary, we investigated whether increasing arousal with constant valence affects time perception and whether this is reflected in an ERP that has previously been linked to arousal-based changes in perceptual processing. We showed that EPN amplitude increase correlates with the overestimation of auditory stimuli, when these stimuli are highly arousing. This recommends EPN amplitude as a marker of arousal-based time dilation. We have looked at the arousal effect with a medium-level valence, further studies are needed to see whether EPN shows different trends under different emotional conditions.
